# Preparation of Magnetic Carbon Nanotubes (Mag-CNTs) for Biomedical and Biotechnological Applications

**DOI:** 10.3390/ijms141224619

**Published:** 2013-12-18

**Authors:** Andrea Masotti, Andrea Caporali

**Affiliations:** 1 Gene Expression-Microarrays Laboratory, Bambino Gesù Children’s Hospital-IRCCS, P.za S.Onofrio 4, Rome 00165, Italy; 2 University of Edinburgh, University/BHF Centre for Cardiovascular Science, Queen’s Medical Research Institute, 47 Little France Crescent, Edinburgh EH16 4TJ, UK; E-Mail: a.caporali@ed.ac.uk

**Keywords:** magnetic carbon nanotubes, multifunctional vectors, nanotechnology, nanobiotechnology, biomedicine

## Abstract

Carbon nanotubes (CNTs) have been widely studied for their potential applications in many fields from nanotechnology to biomedicine. The preparation of magnetic CNTs (Mag-CNTs) opens new avenues in nanobiotechnology and biomedical applications as a consequence of their multiple properties embedded within the same moiety. Several preparation techniques have been developed during the last few years to obtain magnetic CNTs: grafting or filling nanotubes with magnetic ferrofluids or attachment of magnetic nanoparticles to CNTs or their polymeric coating. These strategies allow the generation of novel versatile systems that can be employed in many biotechnological or biomedical fields. Here, we review and discuss the most recent papers dealing with the preparation of magnetic CNTs and their application in biomedical and biotechnological fields.

## 1. Introduction

Carbon nanotubes (CNTs) have been widely studied for their potential applications in electronic devices, hydrogen storage, drug delivery systems, adsorption and separation processes [1–3]. However, one of the major drawbacks for their application in the biomedical field is the low solubility of CNTs in aqueous solutions. The difficult manipulation in other solvents limits their applications also in other biotechnological fields. Interestingly, CNTs are promising nanostructures owing to their ability to move among different body’s compartments/tissues and to penetrate easily into cells. Furthermore, their intrinsic stability in the biological environment coupled to a high surface area and an internal open space to be filled with therapeutic drugs are among the most attractive properties [4–7].

It has been reported that properly functionalized CNTs display low toxicity *in vivo* even at relatively high concentrations [4]. The surface functionalization of CNTs with metallic nanoparticles has led to the preparation of powerful nanohybrids successfully employed not only in catalysis, gas sensors, and fuel cells [8–11] but also for biomedical imaging, biomanipulation, supercapacitor, and environmental treatments [12–16]. In fact, these nanomaterials have a huge potential as contrast agents for MRI [17], catalysis [18], magnetic hyperthermia [19] and in data storage devices [20] and the magnetic delivery of CNTs through an external magnetic field is considered a promising approach to achieve specificity (targeted delivery) when directing these nanosystems to diseased organs [21]. On the other hand, CNTs possess also a hollow cavity that can be filled with a variety of metals such as Au, Ag, Cu, Sn, Fe, Co, and Ni and employed as nanoantennas or microscopic probes [22–24]. For these reasons, many studies increasingly focus interest in functionalization (or coating) of CNTs (*i.e.*, with magnetic or superparamagnetic nanoparticles) or in filling their cavity with magnetic molecules in order to obtain versatile systems able to be employed more efficiently in biomedical or bioimaging applications. In this review, we will discuss the most widely employed techniques to obtain magnetic CNTs (Mag-CNTs) and their applications especially in the biomedical and biotechnological fields.

## 2. Preparation of Magnetic Carbon Nanotubes

The chemical combination of magnetic nanoparticles or nanocrystals and CNTs in order to obtain nanohybrid structures, follows various stratagies: encapsulation of magnetic molecules inside the carbon nanotubes (endohedral functionalization) or grafting/decorating CNTs on their surface (exohedral functionalization) by bioconjugation chemistry or electrochemical deposition. The strategies to attach ready synthesized nanocrystals have been achieved using covalent bonds [25], electrostatic interactions [26], π–π stacking [27], and hydrophobic interactions [28]. Many other strategies have been devised in the last few years and we will discuss separately their applications, according to their different preparations.

### 2.1. Carbon Nanotubes Filled with Metals

The first attempt to fill CNTs with metals is represented by the preparation of monocrystalline FeCo nanowires encapsulated inside multiwalled carbon nanotubes, recently reported by Elias *et al.* [29]. These nanowires are not subjected to oxidation owing to the presence of the insulating carbon nanotubes. The preparation of these nanowires consists in the aerosol thermolysis of ferrocene and cobaltocene solutions in toluene under inert atmospheres. In particular, the solutions have been atomized and pyrolyzed at temperatures varying from 600 to 800 °C. The characterization of these systems demonstrates the homogeneous concentration of Fe and Co (monocrystals) inside the CNTs, assessing their enhanced mechanical properties. The metallic surface of these nanowires is not oxidized due to the presence of an insulating carbon nanotube layer. The resulting FeCo nanowires also display large coercive fields at room temperature (e.g., 900 Oe), thus representing optimal starting materials for the fabrication of high-density magnetic storage devices, magnetic power generating systems (operating at high temperatures under high mechanical stress) and other magnetic composites. Unfortunately, no applications in the biomedical or biotechnological field have yet been reported.

**Figure 1.**
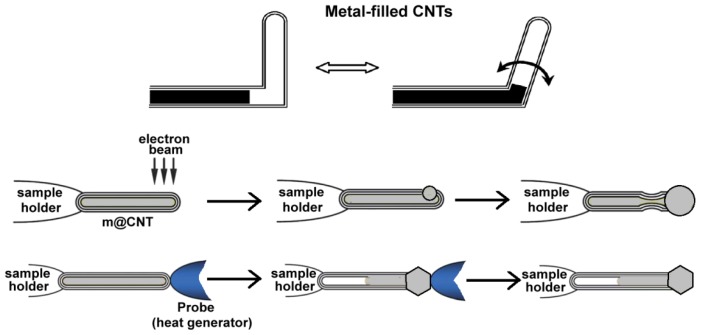
CNTs filled with metals. Two methods for bubbling metals from the nanotubes (electron beam focusing and heat generation) in order to obtain metallic nanotips/nanoantennas are illustrated.

The hollow cavity of carbon nanotubes can also be filled a variety of metals, such as Ti, Cr, Fe, Co, Ni, Cu, Ga, In, Zn, Mo, Pd, Ta, W, Gd, Dy, Yb, Sn, Hg, and Fe–Co (see references cited in [30]), thus obtaining novel structures with different properties that can be applied in nanoelectronics and nanoelectromechanical systems (*i.e.*, nanoextruders, electrical nanocables, nanomagnets, nanoswitches, nanothermometers, and nano test tubes) (Figure 1) [30]. Generally, the synthetic route is the use of the metal or the metal oxide as the catalyst for the CNT growth. The catalyst is introduced into a hot furnace (>600 °C) previously conditioned with N_2_ gas. Acetylene was introduced into the furnace to form CNTs. With this one-step CVD method, high-yield amorphous flexible CNTs filled with β-Sn nanowires has been efficiently synthesized.

One of the most interesting characteristics of these systems is the possibility to induce deformations to the carbon shell by a simple exposure to an electron beam. This induced deformation indicates that these nanosystems are more flexible than the normal graphite CNTs and might represent a potential source to create nanorelays, nanogrippers, nanoswitches, or nanomanipulators.

### 2.2. Endohedral Functionalization of CNTs

**Figure 2.**
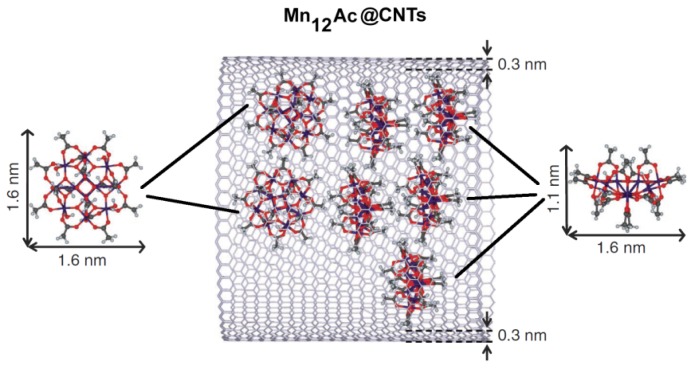
Chemical structure of Mn_12_Ac single-molecule magnets (SMM) and schematic representation of its encapsulation in a carbon nanotube.

One of the methods employed to obtain Mag-CNTs is the encapsulation of magnetic molecules, such as single-molecule magnets (SMM), into the cavity of carbon nanotubes (Figure 2) [31]. The authors employed graphitized multi-walled CNTs sufficiently wide for insertion of SMM and completely free of residual catalysts to avoid interference during the analysis of the magnetic properties of the novel compounds. As starting materials, they used CNTs with a length of 10–50 μm and a mean internal diameter of 6.5 ± 1.8 nm produced by catalytic chemical vapour deposition at 2800 °C and the dodecanuclear mixed-valence manganese carboxylate (Mn_12_O_12_(O_2_CCH_3_)_16_(H_2_O)_4_) (or Mn_12_Ac) [32]. To encapsulate the SMM, CNTs were pre-treated with concentrated nitric acid to open the CNTs and obtain nanotubes with an average length of 400 ± 200 nm. Then, supercritical CO_2_ (scCO_2_) was employed (40 °C, cycling pressure between 120 and 275 bar for 20 h) for the transport of the SMM molecules into the nanotubes due to the small size of scCO_2_, its low viscosity, high diffusivity and zero surface tension that allows it to penetrate the nanotubes without hindrance, enabling the insertion of the desired guest species and the production of the hybrid material Mn_12_Ac@CNT.

This hybrid material was further characterized by thermogravimetric analysis (TGA) and differential scanning calorimetry (DSC) whereas the magnetic and magnetoresistance properties of the SMM have been characterized by superconducting quantum interference device (SQUID) and DC conductivity measurements [31].

One of the most appealing properties of this hybrid compound is the synergistic coupling of the functional properties of nanotubes to the magnetic properties of SMM and the resulting non-covalent interactions responsible for efficient transport and encapsulation of the guest molecules into nanotubes.

In the field of photothermal nanomaterials, a contactless method, based on the local excitation induced by a laser beam, generated cobalt cluster-filled carbon nanotubes [33,34]. This technique exploited the thermal properties of CNTs and the gradients that can be established within the tube itself. In previous works, the direct use of thermal gradients to induce mass transport (a process called thermophoresis) allowed the manipulation and control of flow through the nanodevice, driving liquids (*i.e.*, water) as nanodroplets inside single and double-walled nanotubes [35].

Similar results have been obtained by Barreiro *et al.* who fabricated an artificial nanomotor applying a current of 0.1 mA to a multi-walled CNT, thus inducing an electrically-induced thermal gradient, which was able to move a cargo along the nanotube [36]. Unfortunately, the application of these compounds in the biomedical field has not been reported yet.

**Figure 3.**
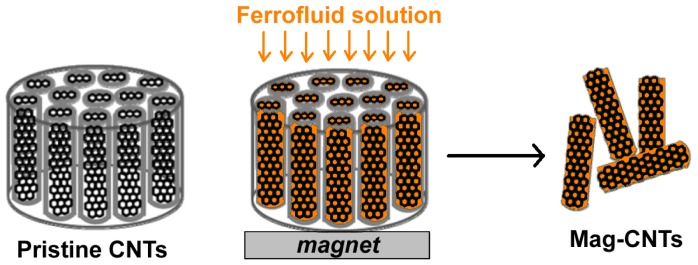
CNTs grown within the pores of an alumina template are filled with magnetite nanoparticles by exploiting the magnetic field generated by placing a magnet beneath the assembly. After filling, CNTs can be easily recovered for downstream applications.

CNTs can be also filled with ferrofluids, such as superparamagnetic iron oxide nanoparticles (Fe_3_O_4_), and employed as magnetic sensors. Korneva *et al.* proposed that these magnetically filled CNTs can potentially be used as nanosubmarines driven through blood vessels by an external magnetic field for transporting drugs to specific locations in the body, as well as for medical diagnosis without surgical interference [37]. Usually, the filling of CNTs with monodisperse Fe_3_O_4_ nanoparticles is a challenging task. Recently, a multistep process to obtain CNTs filled with superparamagnetic nanoparticles, also called “nanostraws”, has been reported (Figure 3) [38]. Carbon nanotubes have been grown by CVD within the pores of an alumina template, whereas the magnetite nanoparticles have been prepared by standard chemical synthesis (hydrothermal reaction starting from iron acetyl-acetonate, Fe(acac)_3_). The magnetic nanoparticles have been incorporated inside CNTs employing a magnetically assisted capillary action method in order to produce CNTs completely filled with uniform particles of Fe_3_O_4_[37].

In particular, CNTs are open at both ends and fixed in an alumina template that has been placed over a permanent magnet. A suspension of Fe_3_O_4_ in hexane was poured drop-wise on the top of the nanotubes’ opening. The solution penetrated deeply into the nanotubes by both capillarity action and magnetic field, which helped to increase the amount of magnetic nanoparticles loaded into CNTs. After evaporation of the solvent, CNTs have been cut into small pieces and properly purified. The authors demonstrated that these systems have good magnetic properties owing to the increased dipolar interparticle interactions. These magnetic CNTs may represent good candidate systems for magnetic-field guided applications.

### 2.3. Exohedral Functionalization of CNTs

#### 2.3.1. Decoration of the Carbon nanotubes’ Surface with Magnetic Nanoclusters

**Figure 4.**
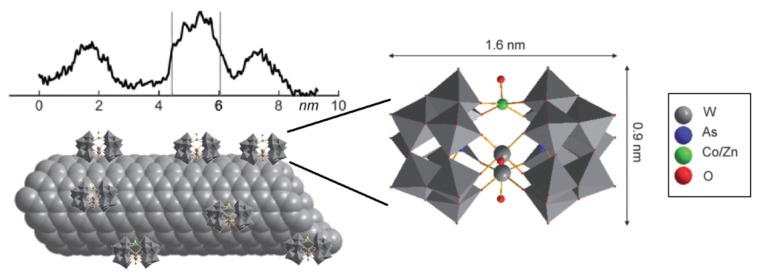
Structure of magnetic metal polyoxometalate (MPOM) cluster attached to CNTs. The representation of the CNT profile of density (obtained by HAADF-STEM) clearly emphasize the presence of several clusters attached along the outer surface.

A method to obtain magnetic carbon nanotubes by assembling a magnetic polyoxometalate (POM) encompassing a single cobalt ion (CoPOM, (As_2_W_20_O_68_Co(H_2_O))^8−^) or its isostructural diamagnetic zinc analogue (ZnPOM, (As_2_W_20_O_68_Zn(H_2_O))^8−^) to the surface of a carbon nanotube has been recently described by Charron *et al.* (Figure 4) [39]. Briefly, CNTs have been added to an acidic solution of the metal POM and the mixture has been stirred in an ultrasonic bath for 20 h at a temperature <12 °C. This led to a dark suspension that was subsequently centrifuged. The supernatant has been isolated, filtered and the filtrate washed with water giving rise to metal polyoxometalate CNTs-Mg. The dimension of the metal POM is approximately 0.9 × 1.6 nm and it is compatible with bulges observed on the surface of the nanotubes by microscopic high-angle annular dark-field imaging coupled to scanning transmission electron microscopy (HAADF-STEM), electron energy-loss spectroscopy (EELS) and macroscopic (XPS, cyclic voltammetry and SERS) measurements.

Most importantly, the authors demonstrated the preservation of the structural and magnetic properties of the POM molecules upon functionalization and also the existence of electronic communication between the molecules and the nanotubes.

**Figure 5.**
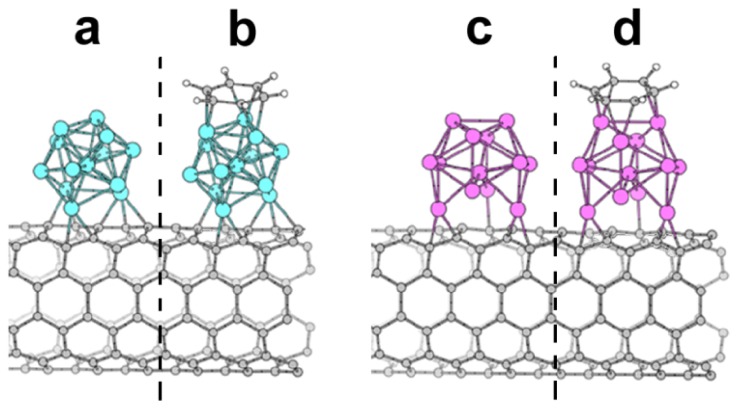
Structure of Ni_13_ (**a**) and Pt_13_ (**c**) clusters attached to CNTs; (**b**) and (**d**) reports the recognition of benzene by these CNTs.

The direct attachment of a metal cluster to the surface of CNTs has been recently reported (Figure 5) [40]. The authors decorated the external walls of single-walled CNTs with metallic clusters (*i.e.*, Ni_13_ and Pt_13_ clusters) that gave rise to a significant magnetization in the CNT. These metallic nanotubes exhibited a very high sensitivity toward gas molecules (*i.e.*, benzene vapors).

The size of the metallic clusters (13 atoms) is the minimum size to impart the cluster with a spherical shape and good energetic stability. Moreover, the magnetic properties are also enhanced (magnetic moment of <0.96 μB/atom for Ni_13_ and up to <0.65 μB/atom for Pt_13_) by the small dimension. Unfortunately, no biological or biomedical applications have been envisaged so far.

#### 2.3.2. Carbon Nanotubes Decorated with Magnetic Nanoparticles

**Figure 6.**
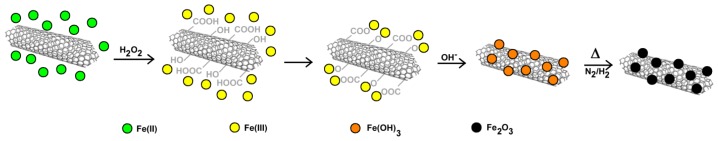
Preparation of magnetic CNTs following the Fenton’s reagent synthetic scheme. The oxidation of Fe(II) to Fe(III) with H_2_O_2_ allows attachment to the oxidized CNTs. A hydrothermal reaction conducted on the whole assembly, under controlled atmosphere, leads to the formation of magnetic CNTs.

Hybrid systems based on iron oxides/carbon nanotubes have many potential applications in electric device, magnetic data storage, and heterogeneous catalysis. The removal of azo dyes is an important issue. In fact, most dyes used in the manufacturing industries contain aromatic rings that are generally toxic or potentially carcinogenic/mutagenic agents [41,42]. With the aim to remove azo dyes (*i.e.*, methyl orange) dissolved in aqueous solution, magnetic CNTs have been also prepared by a straightforward Fenton’s reagent method (Figure 6) [43]. This method consists in the slow addition of H_2_O_2_ to a solution of FeSO_4_, in which CNTs have been suspended. The resulting solution is the so-called Fenton’s reagent.

The oxidant solution not only allows the conversion of Fe(II) to Fe(III) but also the generation of reactive functional groups on the CNT’s surface. Further precipitation of Fe(OH)_3_ followed by heat treatment under a nitrogen/hydrogen flow, produced Fe_2_O_3_ nanoparticles that uniformly dispersed on the surface of CNTs with high loading (>50%). The advantage of this method consists in the preparation of magnetic CNTs without the use of strong acids or exploiting reactions for the formation of covalent bonds. Moreover, this system is able to remove the azo dye methyl orange from aqueous solution by adsorption, to be separated by an external magnetic field and easily regenerated by UV photocatalysis.

Decoration of CNTs by spinel ferrites nanoparticles with the chemical formula MFe_2_O_4_ (M = Mn, Co, Ni, Mg, or Zn) has been reported to improve optical, magnetic and electrochemical properties of pristine CNTs [44–46]. In a recent work, a special electrode has been designed with the aim to determine analytically the concentration of the antibiotic cefixime with voltammetric techniques [47]. Pristine CNTs (diameter of 10–30 nm, length of 5–15 μm) have been treated with HNO_3_ to introduce reactive functional groups then they have been dropped onto the surface of a glassy carbon disk electrode. To obtain magnetic metal functionalized CNTs the authors developed an *in situ* chemical citrate gel method. This procedure consists in the treatment of functionalized CNTs into 1M citric acid followed by addition of a (1:2) solution of Ni/Fe nitrate. The pH was increased to 9 with ammonia and stirred at 30 °C for 48 h to complete the reaction. The substance was finally calcinated at 620 °C for 2 h in argon atmosphere to obtain a powder of NiFe_2_O_4_-MWCNTs. This powder has been supported on a carbon disk electrode and employed in the analytical determination of cefixime (in tablet, blood plasma, and urine samples) by exploiting the presence of its NH_2_ groups that can be oxidized by anodic reactions [48].

**Figure 7.**
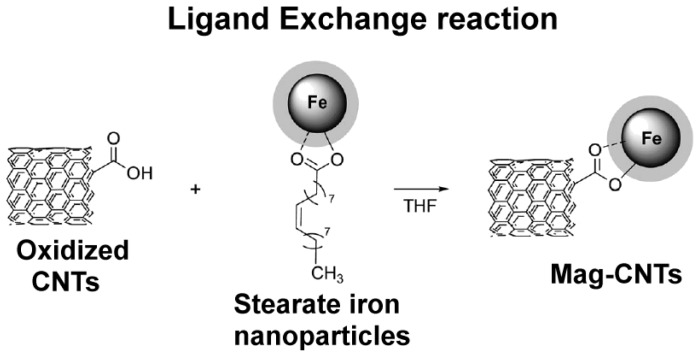
Preparation of magnetic CNTs decorated with magnetic iron oxide nanoparticles (ligand exchange reaction). The magnetic core is exchanged by passing from the stearate molecule to the CNTs.

**Figure 8.**
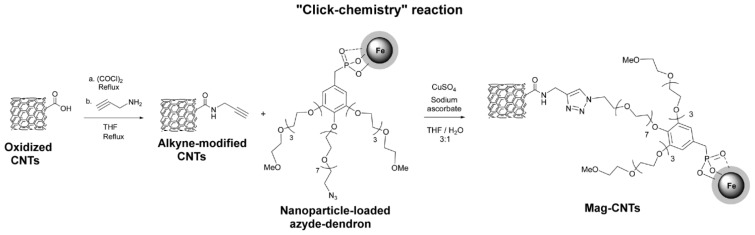
Preparation of magnetic CNTs decorated with magnetic iron oxide nanoparticles (chemoselective ligation or “click chemistry”).

A recent paper dealing with the decoration of CNTs with magnetic iron oxide nanoparticles and exploiting two different reactions (ligand exchange and chemo-selective ligation or “click chemistry”) has been reported (Figures 7 and 8) [49]. The authors suggested that these systems can be employed in applications for cell labeling, MRI cell tracking and magnetic manipulations. First, iron oxide superparamagnetic nanoparticles were synthesized by thermal decomposition of iron stearate in octyl ether and oleic acid. Then, the ligand exchange reaction took place by adding oxidized CNTs (bearing COOH groups) and stirring the suspension in THF for 24 h (Figure 7).

The click ligation has been performed in mild conditions exploiting the Cu(I)-catalyzed azide–alkyne Huisgen 1,3-dipolar cycloaddition reaction in an efficient and highly selective way. CNTs have been functionalized with alkyne moieties, whereas iron oxide nanoparticles have been coated with a dendrimer bearing a terminal azide group. Both derivatized CNTs and nanoparticles have been reacted with sodium ascorbate and Cu(II) sulphate in a THF/H_2_O (3:1) solution (Figure 8).

The degree of functionalization is different for the two reactions owing to the different degree of active groups (COOH and alkyne groups) on the surface of the CNTs. Oxidized CNTs have a greater percentage of substitution but alkyne derivatives favor a more homogeneous derivatization. Moreover, aggregation is reduced when the click chemistry approach is followed. These compounds enter easily into cells, are moderately toxic and display good magnetic properties. These properties can be exploited to monitor these systems by MRI techniques and to manipulate them with magnetic devices. The control of CNTs by external magnetic fields and the monitoring with non-invasive techniques opens new perspectives for targeted therapy or tissue engineering.

**Figure 9.**
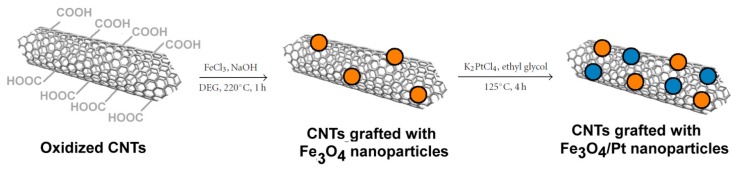
Fe_3_O_4_/Pt nanoparticles loaded on carbon nanotubes (CNTs) by a high-temperature solution-phase hydrolysis method.

In the field of catalysis, CNTs have been exploited as magnetic catalyst support owing to their large surface area and peculiar electronic properties. In particular, Fe_3_O_4_/Pt nanoparticles have been loaded on carbon nanotubes (CNTs) (Figure 9) by a high-temperature solution-phase hydrolysis method [50].

The authors designed a modified synthetic scheme and instead of using the most common acid oxidation method, the oxidation of CNTs has been performed by the nitrene addition method [51]. This method has the advantage of avoiding the use of concentrated acids that can also determine the erosion of the CNT itself, thus maintaining the intrinsic morphology and properties of pristine CNTs. Once the magnetic particles have been formed by the classic hydrothermal reaction (220 °C for 30 min in an alkaline solution), the further attachment of Pt particles has been performed by heating K_2_PtCl_4_ under a nitrogen atmosphere (125 °C for 4 h). These functionalized CNTs can be easily manipulated by external magnetic fields, allowing the recovery and recycling of the catalyst. These CNTs decorated with Fe_3_O_4_/Pt nanoparticles displayed a high catalytic activity in the reduction of 4-nitrophenol to 4-aminophenol and can be readily recycled by a magnet and reused several times. Therefore, these nanosystems are very promising tools in many heterogeneous catalysis applications. In principle, these systems can be also engineered for other biomedical and biotechnological application (*i.e.*, glucose biosensors [52]).

#### 2.3.3. Coating of Magnetic Carbon Nanotubes with Mesoporous Silica

**Figure 10.**
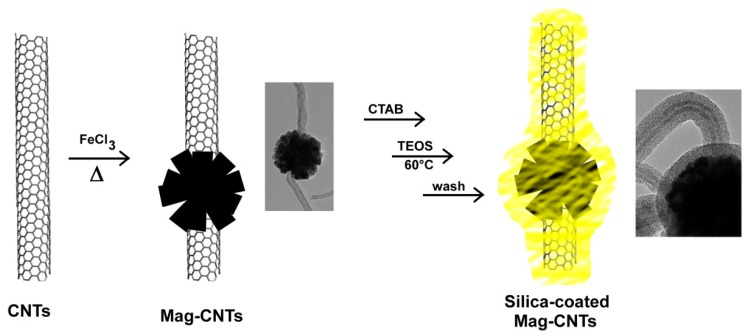
The synthetic route to Mag-CNTs@mSiO_2_.

Another method to obtain exohedral hybrid magnetic carbon nanotubes is the decoration of CNTs with magnetic nanoparticles [44,53–58] followed by coating with mesoporous silica to obtain a magnetic nanocomposite (Figure 10) [59]. The synthetic route to obtain such materials, reported by Lu and colleagues [59], consists in the self-assembly of magnetite (Fe_3_O_4_) nanoparticles along the CNTs (previously treated with HNO_3_ to generate reactive COOH groups on the surface able to bind metal ions) employing a solvothermal reaction. Polyethylene glycol (PEG) was also employed to promote oriented aggregation and avoid uncontrolled crystal growth, whereas sodium citrate was used as capping agent in order to increase solubility in water and ethanol.

After the preparation of Mag-CNTs, the surfactant cetyltrimethyl ammonium bromide (CTAB) has been added to assist the coating of nanotubes with tetraethyl orthosilicate (TEOS). Structural analysis of such nanocomposites by TEM and SEM, revealed that the CNTs were orderly decorated mainly at the end of the nanotube, where the COOH groups’ density is supposed to be the highest. The spherical magnetic particles attached to the nanotube’s surface resulted in a mean diameter of ~200 nm. This nanocomposite material can be easily manipulated by magnetic fields, finding potential useful applications in biomedicine and in the targeted drug delivery field.

**Figure 11.**
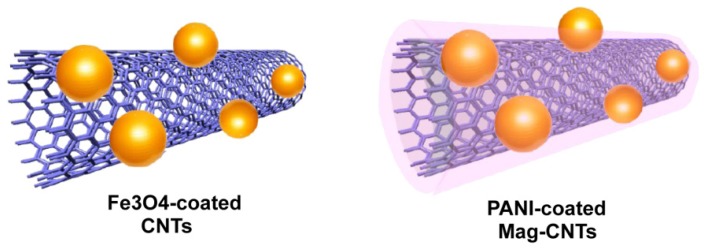
Fe_3_O_4_-coated CNTs (**left**) and PANI/Fe_3_O_4_/CNTs (**right**).

Light-weight nanocomposites filled with carbon nanotubes (CNTs) have been demonstrated to be promising tools in the field of electronics, communication devices, and in the construction of specific parts of aircrafts and vehicles [60–63]. The introduction of magnetic nanoparticles into the nanotubes produces two-phase heterostructures, that can be further coated with polymer molecules to change the final properties of the nanotubes. Recently, a direct morphological comparison between the two-phase magnetic heterostructures (Fe_3_O_4_/MWCNTs) and the three-phase polyaniline-coated Fe_3_O_4_/MWCNTs has been done by Cao *et al.* who conducted also a detailed study on their physicochemical properties (Figure 11) [64].

The Fe_3_O_4_/MWCNT heterostructures were achieved by co-precipitating Fe(II) and Fe(II) metal ions within a dispersion of CNTs. MWCNTs were pre-treated with concentrated sulfuric acid (to add some functional hydrophilic groups) and dispersed in aqueous solution. Addition of NH_4_Fe(SO_4_)_2_ 12H_2_O and (NH_4_)_2_FeSO_4_ 6H_2_O and ammonia led to the production of the desired compound (Fe_3_O_4_/MWCNTs) (Figure 11). To coat the nanostructures with polyamine polymer, the magnetic nanotubes were redispersed in water followed by the addition of aniline and phosphoric acid. In order for the polymerization of aniline to begin, the catalyst ammonium persulfate was added to the nanotubes suspension. The resulting three-phase heterostructures (PANI/Fe_3_O_4_/MWCNTs) were washed (with water and ethanol) and dried in an oven (60–70 °C). SEM, TEM and other techniques were used to characterize the sample. The interface introduced by Fe_3_O_4_ generated resonance in complex permittivity and permeability as well as sufficiently enhanced magnetic loss, resulting in enhancing microwave absorption performance and widening effective absorption bandwidth. The authors suggested that Fe_3_O_4_/MWCNTs are highly effective in electromagnetic shielding and attenuation, but no direct application has been provided.

#### 2.3.4. Coating of Carbon Nanotubes Prior to Incubation with Magnetic Nanoparticles

**Figure 12.**
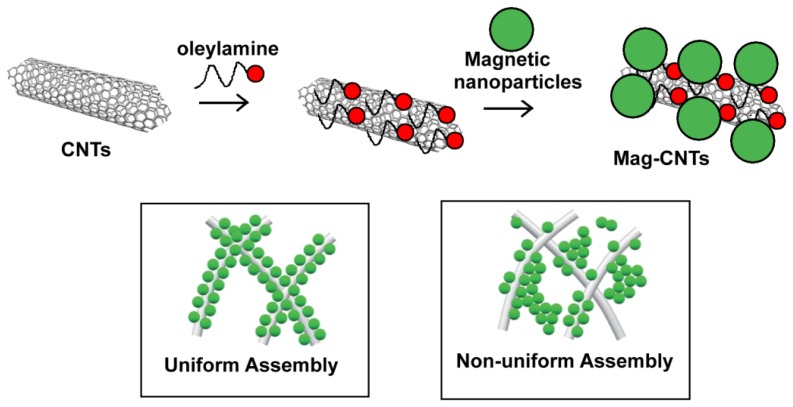
Schematic procedure to obtain CNTs coated with magnetic nanoparticles (Mag-CNTs). Two different assemblies of NPs onto CNTs can be obtained: uniform (**left**) and non-uniform (**right**).

Another method to assemble magnetic nanoparticles on the surface of CNTs without disrupting the pristine nanotube structure has been reported by Li *et al.* [65]. The authors employed a non-covalent chemical approach to prepare the hybrid nanostructure: carbon nanotubes coated with a uniform layer of oleylamine have been sonicated 5 min with maghemite nanoparticles (γ-Fe_2_O_3_) having a mean diameter of 5.7 nm (Figure 12). The resulting compound can be separated magnetically and repeatedly washed prior to use. However, the resulting magnetic nanotubes have the drawback to be soluble only in organic solvents.

Another kind of coating has been reported by Gao *et al.* [13] who reported the preparation of magnetic multiwalled carbon nanotubes (MWNTs) by electrostatic self-assembly of iron oxide nanoparticles on poly(2-diethylaminoethyl methacrylate) (PDEAEMA)-coated nanotubes. The PDEAEMA-grafted MWNTs were reacted with methyl iodide (CH_3_I), which quaternarized the CNTs and allowed the electrostatic binding of superparamagnetic nanoparticles.

The authors employed these systems to manipulate blood cells in a magnetic field. This technique emphasizes the importance of magnetic biomanipulation of cells, genes, and DNA in bioengineering, biomedical therapy, and single-object technology.

#### 2.3.5. Coating of Magnetic Particles with Polymers prior to Incubation with CNTs

**Figure 13.**
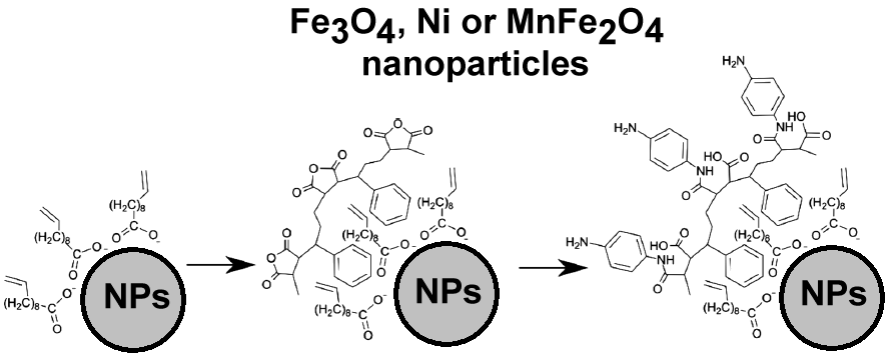
Coating of magnetic particles with polymers prior to incubation with CNTs.

Another means to obtain magnetic carbon nanotubes is the attachment of polymer-coated magnetic nanoparticles to the surface of CNTs previously modified with proper reactive groups (*i.e.*, oxidation with concentrated HNO_3_/H_2_SO_4_ mixtures to obtain COOH groups). Bear and coworkers presented a universal strategy to attach magnetic nanocrystals to the surface of CNTs [66]. The principle they employed is the coating of superparamagnetic nanoparticles (Fe_3_O_4_, Ni, and MnFe_2_O_4_) with the amphiphilic polymer poly(styrene-*co*-maleic anhydride) (PSMA) (Figure 13).

The anhydride rings of the polymer coating have been opened by reacting the nanoparticles with an excess of *p*-phenylenediamine, which introduced reacting amino functionalities on the surface. The reaction of amino-functionalized nanoparticles with carboxy functionalized CNTs through the formation of amide bonds (covalent attachment) produced magnetic CNTs. Moreover, the amphiphilic polymer that covers the nanoparticles, also passivates their surface preventing further reactions. Interestingly, the proposed methodology allows the possibility to obtain nanosystems able to maintain the superparamagnetic properties. This is advantageous when magnetic separation (for purification or delivery purposes) is required. The coating of the nanoparticles before the attachment to the CNTs also prevents the nanotubes from being completely coated by the polymer matrix, which may be undesirable if the nanotubes are used as a support material or further functionalized.

### 2.4. Magnetic Nanoparticles Embedded in the Carbon Nanotube’s Structure

In a recent paper, nearly monodisperse CoFe_2_O_4_ nanoparticles have been deposited on multi-walled carbon nanotubes (MWCNTs) by high-temperature hydrolysis and inorganic polymerization of Co(II) and Fe(III) salts in a polyol solution, affording a novel hybrid nanosystem [67]. This method offers the possibility to extend the preparation of other magnetic ferrite-nanotubes by the use of other metal salts. Briefly, to decorate MWCNTs with CoFe_2_O_4_ nanoparticles, the authors mixed CoCl_2_·6H_2_O and FeCl_3_·6H_2_O (Co/Fe atomic ratio = 2) with oxidized CNTs (conc H_2_SO_4_:HNO_3_ (1:3) at 80 °C for 6 h) into triethylene glycol. The resulting mixture was heated to reflux (250 °C) for 2 h until a black product was formed. The magnetic hybrids have been magnetically separated, repeatedly washed and dried. The percentage of CoFe_2_O_4_ substitution was approximately 60%. Magnetic measurements showed that these hybrids were superparamagnetic at room temperature and their saturation magnetization could be finely modulated by changing the loading of CoFe_2_O_4_ nanoparticles on the nanotubes. The authors suggest that these nanohybrids can be potentially used in the biomedical field as magnetically guided drug delivery systems.

**Figure 14.**
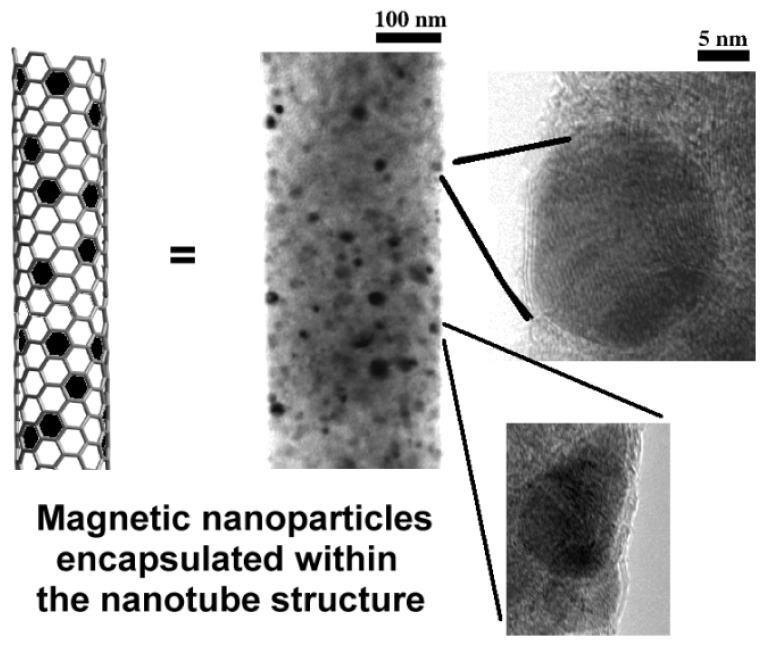
Schematic representation of magnetic nanoparticles encapsulated within the graphitic wall of CNTs and transmission electron microscopy of magnetic CNTs.

Another means to obtain magnetic CNTs without coating the nanotube’s surface with “adsorbent” molecules (*i.e.*, oleylamine) or inserting chemical groups for the conjugation with magnetic nanoparticle has been reported in recent years [68]. The magnetic nanoparticles have been encapsulated within the graphitic wall of the nanotube exploiting the contemporaneous action of template growth and a precursor based on a catalyst/carbon ferrofluid (Figure 14).

The nanoparticles embedded into the nanotube structure are stable even under extreme pH conditions. The particles are distributed throughout the tube length and have an average size of 15 nm. Moreover, this particular procedure allows obtaining nanotubes that can be further functionalized, as the majority of the surface is unaltered, compared to pristine CNTs. This is an advantage over the typical approach of grafting magnetic nanoparticles on the surface of CNTs, or through filling their inner cavity, as the surface is free to accommodate other biological or chemical molecules. This characteristic permits a further functionalization of the graphitic surface with other molecules (*i.e.*, dyes, antibodies, *etc.*) making these magnetic nanotubes promising tools for targeted drug delivery or therapeutic applications. Another advantage is that these modified CNTs have a stronger hydrophilic character, which makes them generally well-suited for biomedical applications. As a potential application of these systems, the drug loading capacity toward diaminophenothiazine (methylene blue) as model drug has been assessed. MB has been used in clinic for several years for the treatment of many diseases [69]. A major drawback of this preparation is the complexity of the synthetic procedure that requires a chemical vapor deposition (CVD) reactors reaching high temperatures (>580 °C), the use of C_2_H_4_ as the external feeding gas and the use of a membranous template on which to grow the CNTs.

## 3. General Discussion about the Preparation of Mag-CNTs

As reported above, the functionalization of magnetic CNTs can be divided into two main strategies: endohedral or exohedral. There are few examples of endohedral preparation in the literature, mainly due to the difficulty in the encapsulation of magnetic materials into a very small nanostructure. The use of supercritical fluids or growing CNTs on specific templates may represent a difficult synthetic strategy for biomedical researchers. Conversely, the exohedral preparation seems more versatile with a different range of polymers used as coating agents and several coupling chemistry adopted for functionalization. This also allows incorporation of drugs within the CNT multifunctional assembly for therapeutic applications. In addition, the exohedral preparation of Mag-CNTs can be exploited also by those researchers who do not have specific chemical expertise or complex instruments for CNTs synthesis. Moreover, pristine CNTs are easily available commercially for most of the applications described in the previous paragraphs.

One of the most discussed aspects when dealing with CNT-based nanostructures is their nanotoxicity and their effects *in vitro* and *in vivo*, regardless of the preparation methods (endohedral or exohedral). Few biomedical studies have been conducted so far, and in our opinion, the endohedral functionalization is less suited for biomedical applications. First, the incorporation of magnetic materials into nanotubes exposes the surface of CNTs to direct contact to the cells, therefore CNTs are not “shielded” and their toxic effects are similar to pristine CNTs. Then, the magnetization efficiency of the incorporated magnetic molecules (or magnets) may be shielded by the nanotube’s layer(s) itself, thus limiting their efficiency. This could lead to a need to increase the dose of the compound, therefore potentially inducing a more pronounced toxic effect. Finally, considerations about the preparation of different Mag-CNT-based systems are separately reported in the following paragraphs where various biomedical applications have been discussed.

## 4. Applications

Functionalized carbon nanotubes have been employed in many fields ranging from biotechnological to analytical biochemistry due to their versatile magnetic properties. Here, we present some interesting applications of multifunctional magnetic CNTs in the biomedical/biotechnological field. For brevity, we summarize only the main findings avoiding a description of those papers where the magnetic properties of CNTs are only due to synthetic impurities, and direct readers to the original papers for more detailed descriptions.

### 4.1. Applications in Biomedicine

In the papers that we have examined and discussed below, magnetic CNTs are generally employed as starting materials for the preparation of more complex multifunctional vectors. These vectors bear fluorescent moieties (*i.e.*, FITC), proteins (*i.e.*, transferrin), and targeting ligands (*i.e.*, folic acid) or are loaded with therapeutic drugs (*i.e.*, doxorubicin). The potential application of multifunctional nanosystems (excluding carbon derivatives such as nanotubes and fullerenes), as novel generators of non-invasive molecular and cellular imaging systems has been reviewed elsewhere [70].

### 4.2. Cellular Imaging

**Figure 15.**
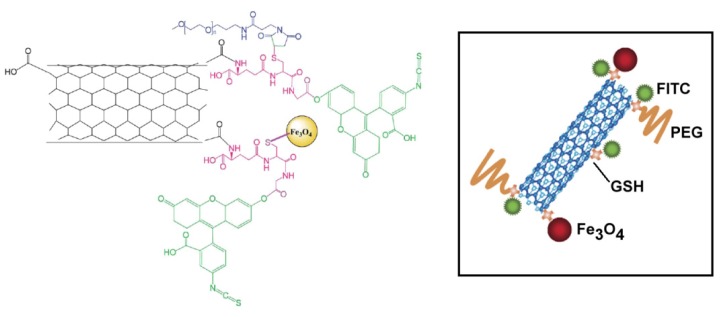
Chemical formula of the Fe_3_O_4_–PEG–FITC–CNT (**left**) and schematic illustration of multicomponent Fe_3_O_4_-PEG-FITC-CNT nanoparticles bound to glutathione (GSH) (**right**).

A multicomponent CNT-based system for cellular imaging applications has been recently reported [71]. In this paper the synthesis of multiwalled CNTs decorated by magnetite nanoparticles (Fe_3_O_4_), poly(ethyleneglycol) (PEG), and a fluorescein isothiocyanate (FITC) dye has been described. The resulting Fe_3_O_4_–PEG–FITC–CNT nanosystem (Figure 15) can be easily suspended in aqueous solution displaying also a good magnetic responsiveness and fluorescent capacity. The Fe_3_O_4_ nanoparticles are grafted firmly onto the surfaces of the CNTs, allowing an easy manipulation of this system by an external magnetic field. Confocal microscopy experiments demonstrated a rapid (2 h) sustained and time-dependent uptake of the multicomponent system at the perinuclear region (but not in the nucleus) of MCF7 cells employed as a model system, without displaying toxic effects. Therefore, this fluorescent Fe_3_O_4_–PEG–FITC–CNT nanosystem has all the properties to represent an ideal candidate for bioimaging, both *in vitro* and *in vivo*. Moreover, as a consequence of the presence of Fe_3_O_4_ nanoparticles attached on the surface, this multifunctional system might be potentially employed in phototherapy and hyperthermia applications through NIR laser radiation and upon application of an external high-frequency magnetic field, as suggested by the authors.

Another application in cancer targeted imaging and magnetically guided drug delivery has been recently reported by Chen *et al.* [72].

**Figure 16.**
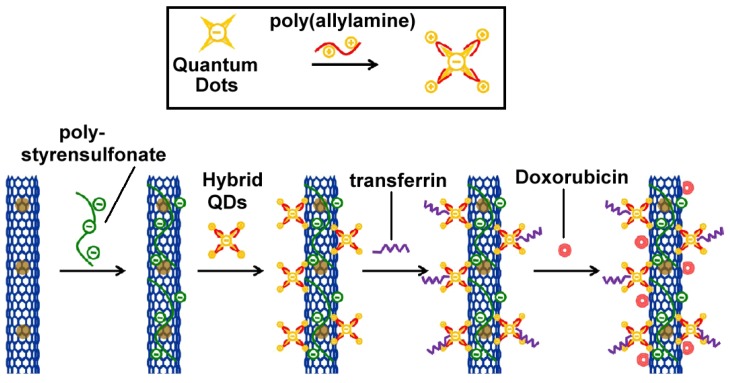
Preparation of water-dispersible DOX-Fe_3_O_4_@CNT-HQDs-Trf conjugates.

In this work, simultaneous cancer-targeted optical imaging *in vitro* and magnetically guided drug delivery has been achieved by developing a multifunctional nanoplatform obtained by conjugating silica-coated CdTe quantum dots with Fe_3_O_4_-filled CNTs. The filling of CNTs with Fe_3_O_4_ facilitates magnetically guided delivery and improves targeting efficiency. Furthermore, the magnetite nanocrystals inside the CNTs protect it from agglomeration, enhance its chemical stability, improve the drug loading capacity (a large effective surface area) and avoid magnetic nanocrystals-induced quenching of fluorescence of the quantum dots. The hybrid SiO_2_ shells of the QDs nanoparticles also mitigate the toxicity of CdTe. By coating the surface of CNTs with transferrin, the authors developed a dual targeted drug delivery system able to effectively transport doxorubicin hydrochloride (DOX) into Hela cells (generally overexpressing the transferrin receptor) by means of an external magnetic field (Figure 16).

### 4.3. Cell Tracking

**Figure 17.**
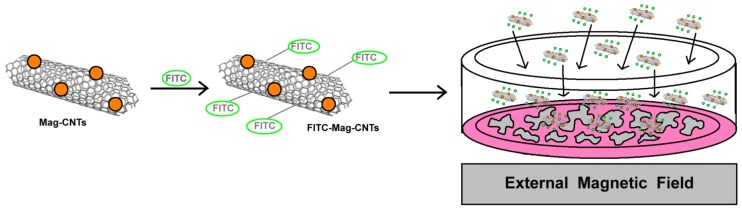
Magnetic field driven magnetic CNTs into HSPCs. Once the magnetic nanosystem has been assembled, the incubation with cells aided by an external magnetic field, improves the transfection efficiency.

Magnetic CNTs can be also employed to track cells as in the case of haematopoietic stem/progenitor cells [73]. Haematopoietic stem and progenitor cell (HSPC) research has significantly contributed to the understanding and harnessing of haematopoiesis for regenerative medicine. Gul and colleagues developed a magnetic single walled CNT-based nanosystem (with an average diameter of 1.2–1.5 nm and a length of 2–5 μm) exploiting the presence of Ni and Y at the tip of the nanotube [73]. These magnetic CNTs have been already employed in magnetic-field-driven biomolecule delivery with very high efficiency [74]. These systems have been synthesized by a sequential reaction scheme involving the oxidation of CNTs, the reaction with thionyl chloride to introduce a highly reactive COCl group followed by conjugation with 2′-(ethylenedioxy)-bis-(ethylamine) to produce CNTs terminally functionalized with amino groups. The amino groups are then reacted with the fluorescent dye FITC, yielding a FITC-labelled highly water-soluble nanosystem (Figure 17).

In order to track HSPC with these systems, umbilical cord blood or peripheral blood leukapheresis CD34^+^ cells (purity >95% and >91%, respectively) has been isolated and further investigated. Briefly, the procedure involves the seeding of CD34^+^ cells in poly-l-lysine culture dishes and the incubation for 45 min at 37 °C, 5% CO_2_. Then, cells were placed into contact with the multifunctional Mag-CNTs at different concentrations (10, 20 and 40 μg/mL) and placed on an Nd–Fe–B permanent magnet (Figure 17). After 1h the uptake of FITC-Mag-CNTs reached 45% even at the lowest concentration (10 μg/mL) reaching its maximum efficiency (83% FITC uptake) after 6 h. An efficiency of 83%, 90% and 100% has been reached after 6 h employing a concentration of FITC-Mag-CNTs of 10, 20 and 40 μg/mL concentrations, respectively. CD34^+^ cells were still ~98% FITC-positive 48h after the uptake, although degradation of FITC over time has been hypothesized to explain the gradual dye photobleaching. The authors suggested that their novel multifunctional nanosystems are efficiently internalized by HSPC in a time- and concentration-dependent manner, regardless of the HSPC source (umbilical cord blood or peripheral blood leukapheresis) and suggested their further use also in other cellular systems (*i.e.*, MCF-7 and difficult-to-transfect THP-1 cells) with the aid of an external magnetic field.

### 4.4. Lymphatic Targeting

The lymphatic system is an important player in the defense against infectious diseases. Lymphatic cancer metastasis occurs frequently even after extended lymph node dissection [75]. No efficient therapeutic methods have been so far developed to target lymphatic metastasis [76]. Therefore, the delivery of chemotherapeutic drugs to the lymph nodes may be of great interest. Nanoparticles can be effectively taken up into lymphatics, but only few nanosystems can be retained in the draining lymph node. Yang *et al.* filled the inner cavity of magnetic nanotubes with chemotherapeutics (*i.e.*, 5-fluorouracil and cisplatin) and employed a magnet to guide the drug matrix to the regional targeted lymph nodes [77]. The nanotubes were retained in the draining lymph nodes for several days and chemotherapeutic drugs were continuously released, allowing a selective killing of tumor cells.

### 4.5. Cancer Lymph Node Metastasis Treatment

A similar application for cancer lymph node metastasis treatment was also reported by Yang *et al.* [78]. The authors used multi-walled carbon nanotubes (40–60 nm) coated with poly(acrylic acid) to prepare magnetic CNTs (by incubation with Fe_3_O_4_ iron oxide nanoparticles). Magnetic nanotubes were further incubated with gemcitabine affording a product with a drug loading of about 62%. The human pancreatic cancer cell lines BxPC-3 and SW1990 have been used in this study. Results indicate that magnetic nanoparticles enhance the *in vivo* therapeutic effect of gemcitabine, allowing for a more effective means of holding the chemotherapeutic drugs at the regional metastatic lymph nodes, and an opportunity to treat cancers locoregionally without systemic toxicity.

**Figure 18.**
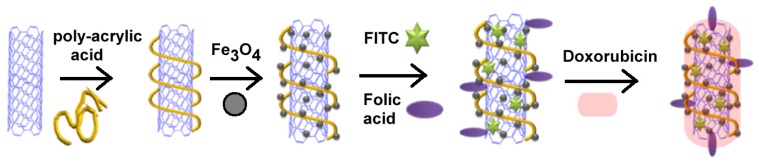
The preparation of Mag-CNTs with targeting properties involves several steps: coating of CNTs with a polymeric material, attachment of iron oxide nanoparticles to the polymer, fluorescent probe (FITC) and targeting molecules (folic acid) attachment and finally coating with doxorubicin.

A magnetic dual-targeted nanocarrier for drug delivery has been developed recently by Lu and coworkers who combined the advantages of Mag-CNTs with the targeting properties of folic acid ligand to convey the anti-cancer drug doxorubicin to cancer cells (Figure 18) [79].

This system can be guided by an external magnetic field and can exploit ligand-receptor interactions to increase drug delivery. In fact, at low pH values (*i.e.*, in intracellular endosomes), doxorubicin is released efficiently from the internalized nanocarriers in the cytoplasm and enters the nucleus to induce a selective killing of U87 cells. As a result of their peculiar properties, these nanovectors have been suggested as novel vehicles to enhance the efficiency of cancer therapy *in vivo*.

### 4.6. Human Monocytic Cells: Implications for Cell-Based Cancer Gene Therapy

Monocyte-based gene therapies in cancer have been hampered by either the resistance of these cells to non-viral molecular delivery methods or poor trafficking to the tumor site after *ex vivo* manipulations [80,81]. Therefore, the cellular uptake efficiency and cytotoxicity of magnetic carbon nanotubes labelled with fluorescein isothiocyanate (FITC) (FITC-Mag-CNTs) to human monocytic leukemia cells (THP-1 cell line) has been investigated for possible application in cell-based gene therapy against cancer [82]. The authors demonstrated that the uptake of FITC-Mag-CNTs into THP-1 cells is 100% effective (1 h after the delivery) and that these engineered CNTs entered the cell cytoplasm and the nucleus, without compromising cell viability. Therefore, the proposed technology could be employed not only to enhance monocytes trafficking to the tumor site after their *ex vivo* manipulations but also to improve gene delivery into monocytes or other hard-to-transfect cells including leukemic cells thus enhancing cell-based cancer gene therapies.

## 5. Conclusions

The papers discussed in this review emphasize the role of magnetic carbon nanotubes (Mag-CNTs) as novel and promising drug delivery vectors for applications in biomedical and biotechnological applications. The synthesis or the preparation of multifunctional nanovectors is nowadays feasible and sometimes straightforward to obtain and will surely lead to increase in the number of *in vitro* and *in vivo* experiments employing these multifunctional nanovectors in the future. Hopefully, novel properties and improved performances of Mag-CNTs will be proposed that will satisfy the ever increasing need of safer and more efficient drug delivery vectors. It is difficult to envisage all the possible applications that will be developed in the future, but the potential of these systems is very high and the range of applications involves several biomedical and biotechnological fields. In the biomedical field, the most promising approach is the development of non-cytotoxic vectors with targeting ability exploited by the presence of magnetic nanoparticles that can be guided through selected tissues by an external magnetic field. Additionally, the presence of magnetic particles can encourage the use of Mag-CNTs as versatile systems for hyperthermia therapeutic strategies, still not completely developed and widespread. We have no doubt that the future for the “black” magnetic carbon nanotubes will be very bright.
